# Engineering Charge Transport by Tunneling in Supramolecular Assemblies through Precise Control of Metal–Ligand Interactions

**DOI:** 10.1002/smll.202501303

**Published:** 2025-05-23

**Authors:** Hungu Kang, Abdalghani H. S. Daaoub, Sara Sangtarash, Jiung Jang, Kangsik Lee, Hatef Sadeghi, Hyo Jae Yoon

**Affiliations:** ^1^ Department of Chemistry Korea University Seoul 02841 South Korea; ^2^ Device Modelling Group School of Engineering University of Warwick Coventry CV4 7AL UK

**Keywords:** assembly, charge transport, coordination, ligand, supramolecular

## Abstract

Coordination‐driven supramolecular assemblies are promising for nanometer‐sized electronic devices due to the potential to manipulate metal–ligand interactions and thereby control charge transport via tunneling through these assemblies. Cross‐plane charge tunneling is investigated in assemblies of metalloporphyrins and pillar molecules, specifically palladium(II) and zinc(II) octaethylporphyrin (PdOEP and ZnOEP) monolayers and bilayers with bidentate (DABCO) and monodentate (ABCO) pillar ligands on highly oriented pyrolytic graphite (HOPG). Junction measurements and quantum‐chemical calculations reveal that metal–ligand interactions significantly influence charge transport via tunneling and thermoelectric effects. Weak interactions in PdOEP assemblies create isolated molecular orbitals on interior pillar ligands, compressing the HOMO‐LUMO gap and enhancing tunneling currents with unusual, inverted attenuation behavior and high thermopower. Conversely, strong interactions in ZnOEP assemblies induce localized orbitals on the porphyrin, leading to conventional tunneling decay behavior and low thermopower. The study highlights the potential of metal–ligand interactions as a strategy to engineer molecular orbital distribution, enhancing quantum transport efficiency in molecular‐scale devices.

## Graphical abstract

Tuning metal–ligand interactions in supramolecular assemblies using metalloporphyrins and axial ligands modulates charge transport. Strong ZnOEP‐ligand bonds show conventional tunneling, while weaker PdOEP‐ligand interactions yield enhanced conductance, negative attenuation, and high thermopower. These results highlight coordination chemistry as a key design principle for optimizing electronic and thermoelectric performance in molecular devices.

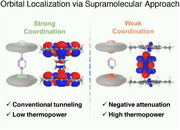

## 1 Introduction

Supramolecular electronics, which leverages diverse non‐covalent interactions such as host‐guest,^[^ ^1^, ^2^ ^]^ *π–π*,^[^ ^2^ ^]^ hydrogen bonding,^[^ ^3^ ^]^ and σ–σ interactions^[^ ^4^ ^]^ to create unique charge‐transport channels, has emerged as a promising avenue for next‐generation electronic devices.^[^ ^5^ ^]^ Recent studies have demonstrated that rationally designed supramolecular structures can enhance electrical conductance,^[^ ^6^ ^]^ enable finely tunable quantum interference,^[^ ^2^, ^7^ ^]^ facilitate long‐range charge transport over tens of nanometers,^[^ ^8^ ^]^ and modulate the work function of metal substrates.^[^ ^8^ ^]^ Supramolecular electronics offers unique functionalities that traditional molecular electronics, relying on covalent small molecules, cannot achieve. For example, small molecules such as oligothiophene can undergo ring rotation within a junction. In contrast, supramolecular junctions, formed through *π–π* stacking interactions between oligothiophene molecules, create a larger twist barrier, resulting in reduced strain distribution and providing stable electrical measurements under external mechanical oscillation.^[^ ^9^ ^]^ The ability to create large, complex, hierarchical structures by assembling smaller molecules through non‐covalent interactions enhances understanding of charge transport properties in biological systems with secondary and tertiary structures.^[^ ^3^, ^10^ ^]^ To harness supramolecular architectures as active components in electronic devices, it is crucial to investigate how manipulating weak non‐covalent interactions influences charge transport behavior.^[^ ^5^, ^8^ ^]^

However, most supramolecular junctions in literature rely on strong metal–ligand bonds,^[^ ^8^, ^11^ ^]^ *π–π* stacking,^[^ ^12^ ^]^ and hydrogen bonding.^[^ ^3^, ^10^ ^]^ In contrast, utilizing low‐coordinate metal centers to deliberately weaken metal–ligand interactions can provide a versatile strategy for tuning charge transport and enabling more flexible molecular electronic design.

Charge transport across an electrode‐molecule‐electrode junction can be described by a transmission function, *T*(*E*), based on single Lorentzian‐shaped energy level.^[^ ^13^ ^]^

(1) $$T\;\left(E\right)=\frac{\Gamma^{2}}{{\left(E-E_{MO}\right)}^{2}+\;\Gamma^{2}/4}$$

This function indicates that the tunneling probability is governed by the energy offset (∆*E*) between the Fermi level (*E*_F_) and the accessible molecular orbital (*E*_MO_), and by the broadening of the molecular orbital (*Г*). Combining the transmission function with the Mott formula explains the thermopower of the molecular junction:

(2) $$S={\left.\frac{\pi^{2}k_{B}^{2}T}{3e}\frac{\partial \ln T\left(E\right)}{\partial E}\right|}_{E=E_{F}}$$

where *S* is the Seebeck coefficient, *k_B_* is the Boltzmann constant, *T* is the junction temperature, and *e* is the electron charge. Tunneling current and the Seebeck coefficient each provide distinct insights into the energy landscape of a molecular junction. Electrical current measurements, analyzed via the Landauer formula, reveal the extent of *T*(*E*)’s overlap with the bias window. In contrast, thermopower measurements inform us about the gradient of ln(*T*(*E*)) at *E*_F_. While these measurements independently provide partial views of the energy topography, their complementary nature allows for a more precise determination of the electronic structure and the mechanisms of molecular charge transport.

**Figure 1:**
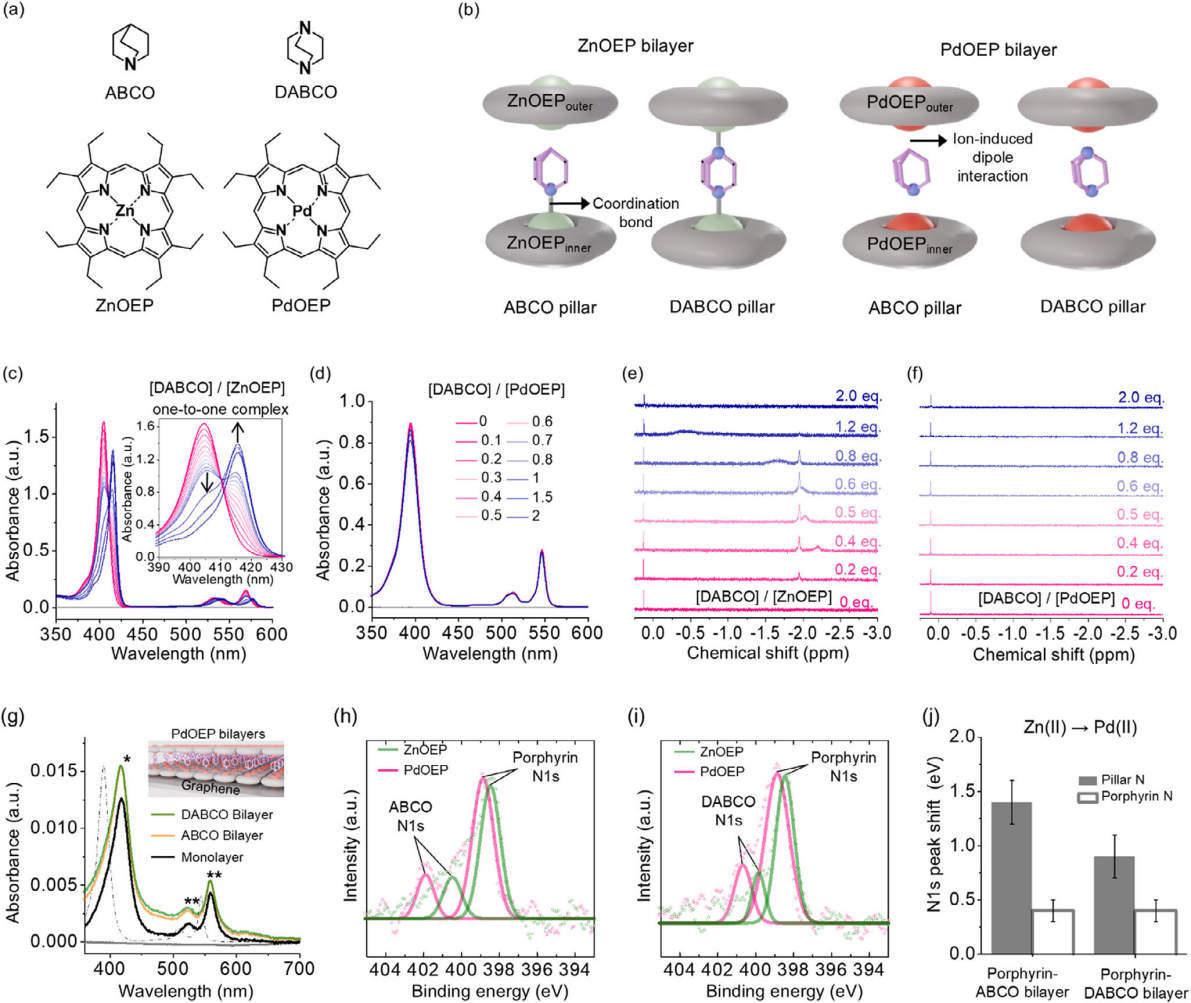
a) Chemical structures of molecules used in this study. b) Structural representation of metalloporphyrin (PdOEP and ZnOEP) bilayers with two different mono‐ and bidentate pillar ligands, ABCO and DABCO. c,d) UV–vis titration experiments were conducted with a fixed concentration of metalloporphyrin solutions (1 × 10^−5^ m in toluene) while varying the DABCO concentration. The molar ratios of DABCO to metalloporphyrin are 0.0, 0.1, 0.2, 0.3, 0.4, 0.5, 0.6, 0.7, 0.8, 1.0, 1.5, and 2.0. e,f) ^1^H NMR titration experiments displaying the spectral changes with varying molar ratios of metalloporphyrin and DABCO in CDCl_3_. g) Solid‐state structural analysis via UV–vis absorption spectroscopy, comparing the absorption spectra of PdOEP monolayer and bilayers with different pillar molecules (DABCO and ABCO) on single‐layer graphene. The dotted line represents the spectrum of pure PdOEP solution as a control. Single and double asterisks indicate the B band (380–500 nm) and the Q band (500–700 nm) transitions, respectively.^[^ ^21^ ^]^ The inset provides a schematic of the PdOEP‐ABCO bilayer structure. h,i) Comparison of high‐resolution X‐ray photoelectron spectra of the nitrogen (N1s) core‐level between ZnOEP and PdOEP bilayer structures with ABCO or DABCO pillars. j) Variation in N1s peak shifts of the pillar part (filled bar) and porphyrin part (blank bar) as the metal center of Zn(II) is substituted to Pd(II).

In this study, we demonstrate that significant variations in metal–ligand bonding strength in supramolecular assemblies influence the distribution and alignment of frontier molecular orbital energies, leading to notable changes in tunneling attenuation and thermoelectric behavior. Specifically, we investigate cross‐plane charge transport in coordination‐driven supramolecular assemblies based on metalloporphyrin and pillar molecules. We formed palladium(II) or zinc(II) octaethylporphyrin (PdOEP or ZnOEP) monolayers and bilayers with bidentate (1,4‐diazabicyclo[2.2.2]octane, DABCO) or monodentate (1‐azabicyclo[2.2.2]octane, ABCO) pillar ligands on highly oriented pyrolytic graphite (HOPG) (**Figure** **1**a,b). In contrast to ZnOEP, PdOEP is less likely to engage in axial coordination due to the low coordination number and square planar geometry of Pd(II), as supported by ^1^H nuclear magnetic resonance (NMR), UV–vis, X‐ray photoelectron (XPS) spectroscopies, and density functional theory (DFT) calculations. Scanning tunneling microscopy (STM) and atomic force microscopy (AFM) analysis indicate that despite the weaker metal‐pillar ligand interaction, the PdOEP bilayer successfully assembled on the substrate. Junction measurements using scanning tunneling spectroscopy (STS) and the eutectic Ga‐In (EGaIn) technique revealed that tunneling current in PdOEP junctions follows the order ABCO‐bilayer > monolayer > DABCO‐bilayer, while ZnOEP junctions exhibit a different trend, monolayer > DABCO‐bilayer > ABCO‐bilayer. Notably, in PdOEP junctions, an unusual feature is observed where the ABCO‐bilayerexhibits a higher current than the monolayer, whereas the DABCO‐bilayer shows a lower current than the monolayer. DFT calculations indicate that in ZnOEP bilayers, the HOMO is localized on the porphyrins, while in PdOEP, it is localized on the pillar ligand due to weaker interactions. This difference in orbital distribution, resulting from slight variations in metal–ligand bonding strength, explains the observed tunneling and thermoelectric behaviors. Our work presents a coordination chemistry‐based strategy for enhancing (thermo)electric performance in molecular‐scale devices.

## 2 Results and Discussion

### 2.1 Metal‐Pillar Ligand Interaction

Zn(II) in ZnOEP prefers an octahedral coordination geometry, forming strong bonds with the nitrogen atoms of ABCO and DABCO pillar ligands.^[^ ^14^ ^]^ In contrast, Pd(II) in PdOEP favors a square planar structure, resulting in weaker interactions with axially approaching pillar molecules. This difference in coordinative bonding allows for the examination of how metal–ligand coordination affects the quantum transport characteristics of supramolecular‐based junctions. We investigated the metal‐pillar ligand interactions between ZnOEP and PdOEP in solution using UV–vis absorption spectroscopy (Figure 1c,d). For ZnOEP, the B band (transition to the second exited state, 380–500 nm) shifted from 406 to 416 nm as the DABCO ratio increased, indicating the formation of a one‐to‐one ZnOEP‐DABCO complex (Figure 1c).^[^ ^14^, ^15^ ^]^ The association constant (*K*_a_) for this complex was determined to be 3.11 × 10^8^ M^−1^ (Figure S1, Supporting Information).^[^ ^14^, ^15^ ^]^ It is noteworthy that the *K*_a_ for two‐to‐one complexation is typically too high to be measured reliably at the concentration levels used in UV–vis spectroscopic analysis;^[^ ^14^, ^16^ ^]^ it can be measured using ^1^H NMR spectroscopy (see below for details). Conversely, the UV–vis absorption spectra of PdOEP remained unchanged with the increasing DABCO ratio, confirming weaker interaction (Figure 1d). These findings were further supported by ^1^H‐NMR spectroscopy. Upon adding 0.5 equivalents of DABCO to ZnOEP, new proton peaks corresponding to complexed DABCO appeared at −1.95 and −2.03 ppm, indicating that each DABCO molecule forms coordination bonds with two ZnOEP molecules. (Figure 1e). With further addition of DABCO, the peak at −2.03 ppm shifted downfield and became broad, suggesting rapid exchange between ZnOEP and DABCO molecules.^[^ ^14^, ^16^, ^17^ ^]^ In contrast, no proton peaks for complexed DABCO were observed in PdOEP solutions, even with added DABCO (Figure 1f), demonstrating that PdOEP barely forms stable coordination bonds with the molecule due to the square planar preference of Pd(II).

While the strong interaction between ZnOEP and pillar ligands ensures the formation of stable bilayer structures on surfaces, it was essential to determine whether the weaker interactions of PdOEP with pillar ligands could produce stable and uniform bilayer assemblies over a large area. To test this, we formed PdOEP bilayers on transparent single‐layer graphene substrates by first physisorbing PdOEP and then treating it with either ABCO or DABCO ligands, following a previously reported method.^[^ ^18^ ^]^ Porphyrin molecules adsorb on graphene or HOPG substrates due to their large planar π‐character, facilitating π‐bonding interaction with the surface.^[^ ^19^ ^]^ This non‐covalent feature allows porphyrins to form well‐ordered monolayers through self‐correction and assembly, resulting in minimal defects.^[^ ^20^ ^]^ We compared the UV–vis absorption spectra of the PdOEP monolayer and bilayers formed on single‐layer graphene substrates.

The bilayer showed an increased peak intensity compared to the monolayer, indicating the formation of a thicker bilayer. However, the peak position of bilayer was identical with that of monolayer, suggesting minimal or weak coordination bonds between PdOEP and the pillar molecules (Figure 1g). The ZnOEP bilayers also exhibited increased B‐band intensity and a ≈2 nm peak shift compared to the monolayer (Figure S2, Supporting Information). The absorbance peaks of PdOEP layers on the surface were red shifted compared to those of PdOEP in solution, with Q band peaks (a weak transition to the first exited state in the range between 500 and 700 nm) shifting from 510 and 544 nm in solution to 525 and 559 nm on surface, respectively. This redshift is attributed to charge transfer from the substrate via *π–π* interactions, rather than coordination with the metal center.^[^ ^22^ ^]^ This conclusion is supported by the 15 nm redhift between the Q band peaks and the absence of new peaks.

XPS analysis revealed differences in the N 1s peak in ZnOEP and PdOEP bilayer structures within the ABCO (Figure 1h) or DABCO (Figure 1i) pillar systems. Upon substituting the metal center from Zn(II) to Pd(II) in the structures, the N1s peaks associated with both the pillar and the porphyrin moiety shifted to higher binding energies (Figures 1h,i). However, the extent of the N1s peak shifts in the ABCO and DABCO pillar molecules (filled bar in Figure 1j) was significantly greater than the shifts observed in the porphyrin moiety (blank bar in Figure 1j). This substantial difference in the N1s shifts for the pillars is attributed to the stronger interaction of the N atom with Zn(II) than Pd(II). In contrast, for the porphyrin N1s, although all configurations maintain coordination bonding with the metal, the minor variations in binding energy (≈0.4 eV) appear to arise simply from differences in the atomic numbers of the metals between Zn(II) and Pd(II), which is similar to the N1s peak shift of the PdOEP monolayer from ZnOEP monolayer (≈0.4 eV) (Figure S3, Supporting Information).

### 2.2 Characterization with STM

To investigate the packing structure of metalloporphyrin assemblies, we conducted STM and AFM analysis under ambient conditions. The STM study for ZnOEP layer, whether in monolayer or bilayer form, exhibited a densely packed structure over a large area (Figures S4–S6 for STM and S9 for AFM, Supporting Information), consistent with previous STM studies on Zn‐porphyrin multilayers.^[^ ^18^, ^23^ ^]^ However, the impact of weaker interactions between metal and pillar molecules on the structure of metalloporphyrin assemblies has been scarcely studied. Therefore, we extensively analyzed the structure of PdOEP bilayers on HOPG.

**Figure 2:**
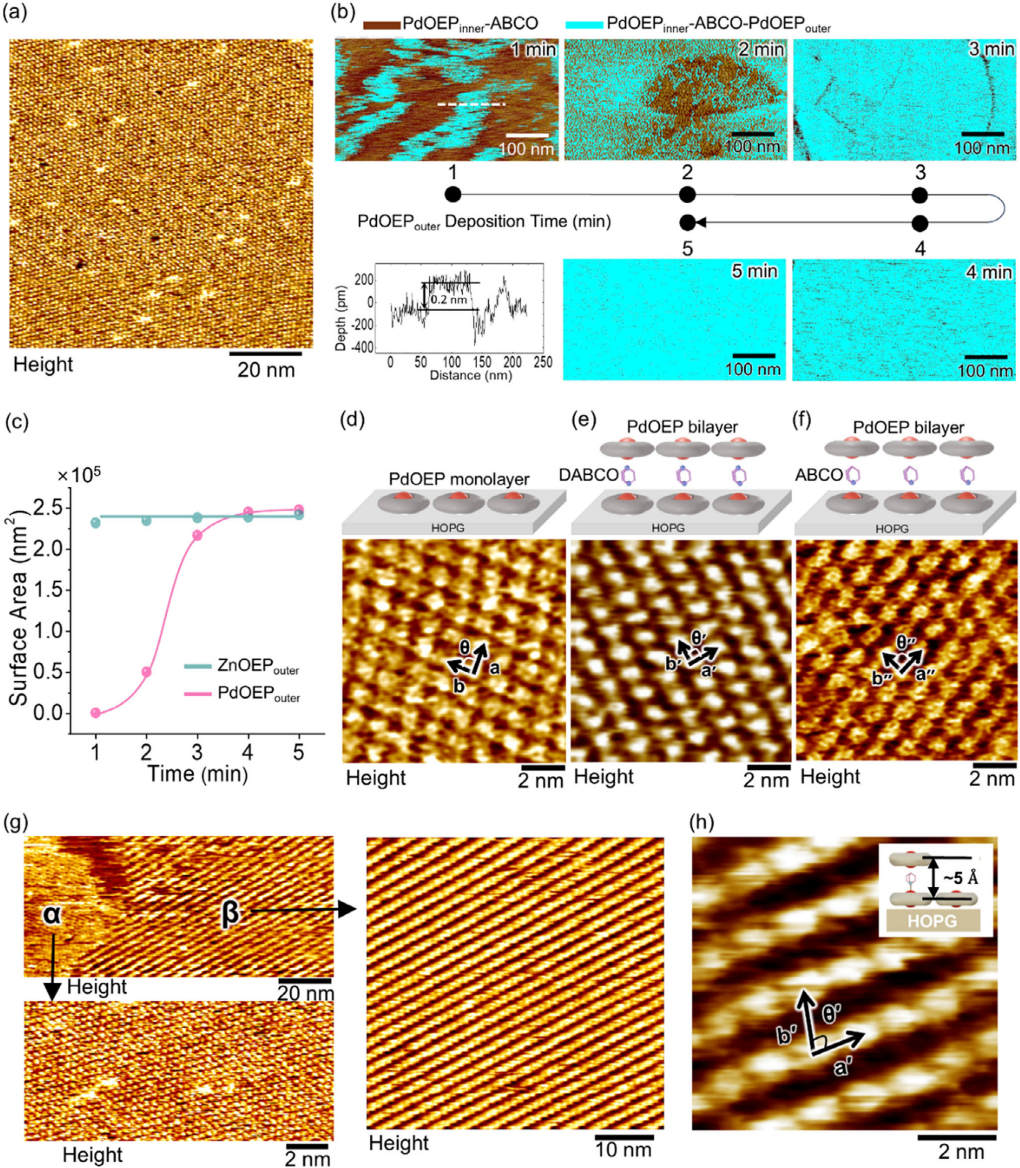
a) Large area (100 × 100 nm^2^) STM image of PdOEP bilayer formed by ABCO pillars. b) AFM analysis showing the changes in surface coverage of the PdOEP_outer_ (cyan color) on the PdOEP_inner_‐ABCO structure (brown color) as a function of the PdOEP_outer_ deposition time. The surface depth profile in the AFM image for 1 min deposition time represents the monolayer thickness of PdOEP (≈0.2 nm) due to the adsorption of PdOEP_outer_ molecules. c) Plots showing the variation in the surface area of the ZnOEP_outer_ and PdOEP_outer_ layers as a function of the deposition time of the porphyrin_outer_ layer. High‐resolution STM images of d) PdOEP monolayer, e) PdOEP‐DABCO bilayer, and f) PdOEP‐ABCO bilayer on HOPG. g) STM images showing two different phase structures (α and β) of PdOEP‐ABCO bilayer on HOPG. (h) Magnified STM image of β‐phase structure.

**Figure** **2**a presents an STM image of a well‐ordered PdOEP bilayer formed by ABCO spacers over a large area (100 × 100 nm^2^). Figure S7 (Supporting Information) also shows a larger‐area (200 × 200 nm^2^) STM image of the PdOEP bilayer with DABCO spacers. This observation reveals that the pillar molecules and the PdOEP_outer_ molecules are not randomly overlaid on the PdOEP_inner_ layer; rather, they are regularly arranged despite the weak metal‐pillar ligand interaction. While the ABCO spacer forms weak coordination bonds with the metal centers of inner and outer PdOEP molecules, the ABCO spacer enables the PdOEP_outer_ molecules to interact via ion‐induced dipole interactions (Figure 1b). This interaction induces a significant charge transfer between the PdOEP molecule and the spacer molecules (Figure S19, Supporting Information). The approach of the Pd(II) ion induces a dipole in the terminal ─CH group of ABCO by disturbing the electron distribution in the nonpolar species.^[^ ^24^ ^]^ Although this ion‐induced dipole attraction is relatively weak, its bonding strength surpasses that of hydrogen bonds and dipole–dipole forces.^[^ ^24^ ^]^ The bilayer exhibited high order and uniformity across most areas, indicating that the weak ion‐induced dipole interaction was sufficient to form an ordered bilayer structure. However, due to the weak interaction between the ABCO spacer and PdOEP_outer_ layer, the bilayer easily desorbed during tip scanning at a fixed point (Figure S8, Supporting Information).

We studied the kinetics of supramolecular assembly over large areas. Surface structure analysis indicated that, initially, the PdOEP_inner_‐ABCO structure was established, followed by a gradual increase in the deposition time of PdOEP_outer_ from 1 to 5 min. AFM images indicate the changes in surface coverage of the PdOEP_outer_ layer (cyan color in Figure 2b) over a large area (500 × 250 nm^2^). After a deposition time of 5 min, nearly full coverage of the PdOEP_outer_ layer was achieved. Figure 2c illustrates plots showing the variation in the surface area of the ZnOEP_outer_ and PdOEP_outer_ layer on the porphyrin_inner_‐ABCO structure, measured via AFM, with varying deposition times. See the AFM data for ZnOEP bilayer in Figure S9 (Supporting Information). Results show that the ZnOEP_outer_ layer achieves nearly full coverage within 1 min of deposition, indicative of strong coordination bonding with the ABCO pillars, leading to a rapid formation of the bilayer. In contrast, the PdOEP_outer_ layer forms gradually over 1 to 2 min, achieving full coverage only after 3 min, reflecting weaker interactions with the ABCO pillars and thus a slower bilayer formation. However, a deposition time of 5 min is also sufficient for forming a complete PdOEP bilayer.

The PdOEP molecules were densely packed in a quasi‐orthogonal lattice arrangement, with detailed lattice structure (a′′ = 1.2 ± 0.03 nm, b′′ = 1.0 ± 0.02 nm, and θ′′ = 85° ± 2°) similar to those for the DABCO bilayer (a′ = 1.2 ± 0.03 nm, b′ = 2.0 ± 0.02 nm, and θ′ = 85° ± 2°) and the monolayer (a = 1.2 ± 0.03 nm, b = 2.0 ± 0.03 nm, and θ = 90° ± 2°) (Figure 2d–f). Both ABCO and DABCO spacers aligned in one axial direction on the monolayer, facilitating the bilayer's adherence to the monolayer structure. Wang et al.^[^ ^25^ ^]^ demonstrated that a multilayer of Sn‐phthalocyanines, created via molecular deposition under UHV conditions without pillar molecules, contained randomly dispersed molecules due to the mobility of the second molecular layer. This highlights the crucial role of pillar molecules in achieving the ordered bilayer structure observed in our experiments.

In the PdOEP‐ABCO bilayer, a stripe structure (defined as β phase) was observed, distinct from the densely packed quasi‐ orthogonal lattice (α phase) (Figure 2g). The β phase, characterized by bright molecular spots, orthogonal lattice (α phase) (Figure 2g). The β phase, characterized by bright molecular spots, exhibited a lattice structure with unit cell dimensions: a′ = 1.2 ± 0.03 nm, b′ = 2.0 ± 0.03 nm, and θ′ = 85° ± 2° (Figure 2h). The depth profile in the β phase showed ≈5 Å of depth, matching the distance between the inner and outer PdOEP layers (Figure S10, Supporting Information). The β phase was a minor component, covering less than 10% of the total area. Its formation mechanism is currently unclear, but we hypothesize that the β phase represents a defect arising from the linear detachment of molecules from the α phase. Notably, the β phase was observed only in the ABCO‐bilayer, likely due to the weaker interaction between the ABCO ligand and the outer porphyrin, and was absent in the DABCO‐bilayer.

### 2.3 Electrical Properties

**Figure 3:**
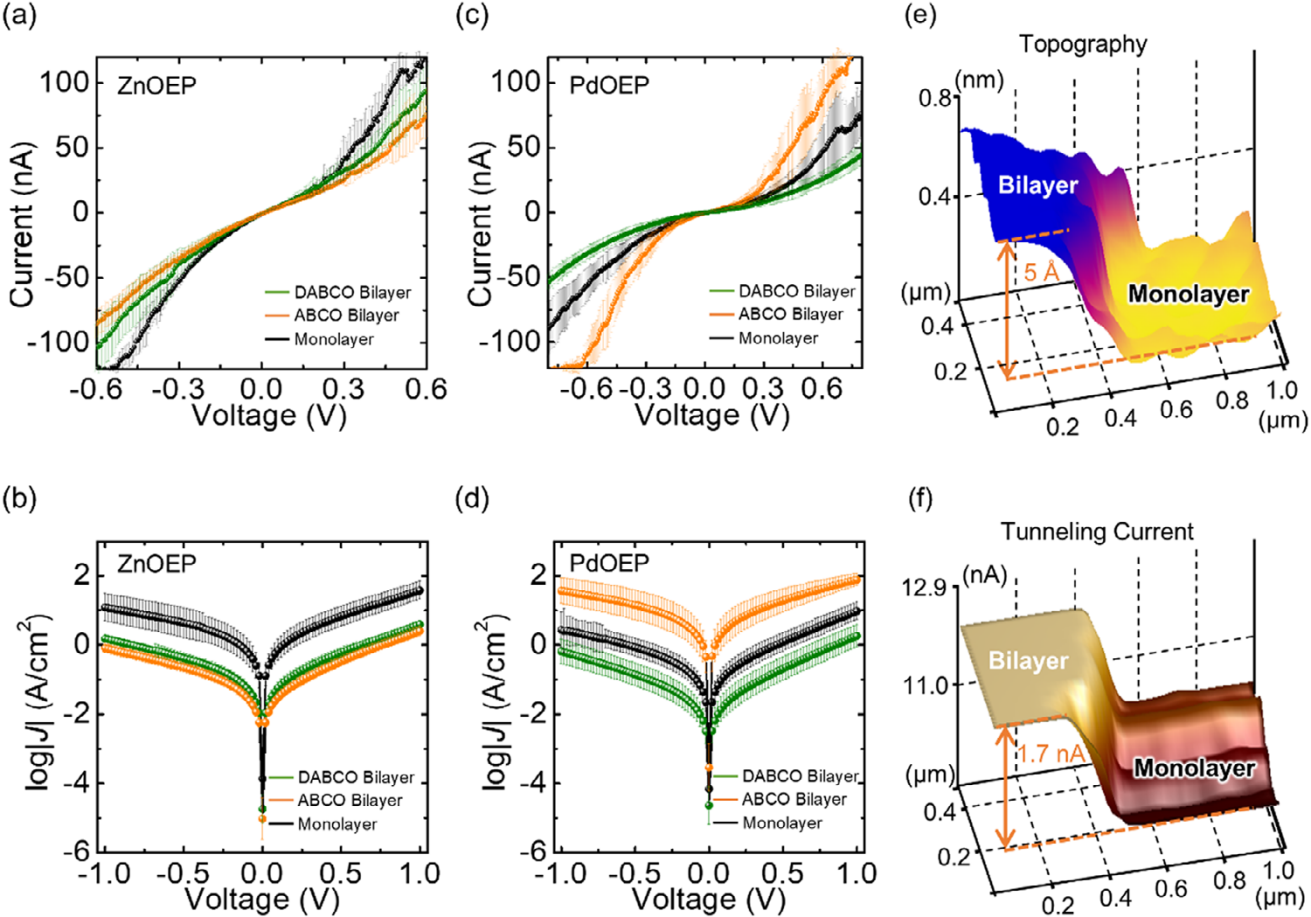
a,c) *I–V* curves of metalloporphyrin (ZnOEP and PdOEP) monolayer and bilayers with DABCO and ABCO pillar ligands, obtained using scanning tunneling spectroscopy (STS). b,d) Log|*J*|*–V* curves of the metalloporphyrin samples, measured using the EGaIn large‐area junction technique. e,f) Conductive probe‐AFM analysis of a defect site at the interface between a PdOEP monolayer and a PdOEP‐ABCO bilayer, simultaneously capturing both 3D‐topography and tunneling current images.

To investigate the electrical characteristics of the porphyrin layers, we employed two techniques: STS for single‐molecule measurements and the EGaIn technique for ensemble‐level measurements. For STS measurements, molecularly resolved STM images were first obtained. The tip was then positioned over individual molecular sites at a distance of 0.5 – 1 nm. With the tip held stationary, current–voltage (*I–V*) curves were recorded by sweeping the bias from −1.0 to + 1.0 V. This process was repeated at ≈20 distinct molecular locations, yielding over 20 *I–V* curves (**Figure** **3**a,c and **Table** **1**). For large‐area junction measurements, ≈400–600 current density–voltage (*J*–*V*) scans were conducted per junction, testing 20–30 different junctions per sample (Figure 3b,d). Each assembly was tested across three to five different samples. In ZnOEP‐based supramolecular assemblies, the mean current at + 0.5 V measured by STS increased in the order of ABCO‐bilayer (59.1 nA), DABCO‐bilayer (74.1 nA), and monolayer (109.3 nA). EGaIn junction measurements revealed a similar trend: the mean values of log|*J*(+ 1.0 V)| were 0.40, 0.59, and 1.58 A cm^−2^ for the ABCO‐bilayer, DABCO‐bilayer, and monolayer, respectively (Figure 3b). The yield of working junctions was 80% for the monolayer, 83% for the ABCO‐bilayer, and 86% for the DABCO‐bilayer. The simplified Simmons equation addresses the length‐dependence of electrical current (or current density) in molecular junctions:^[^ ^26^ ^]^

**Table 1:** Summary of the scanning tunneling spectroscopy (STS) measurements.

Sample	ZnOEP			PdOEP		
	Monolayer	DABCO‐bilayer	ABCO‐bilayer	Monolayer	DABCO‐bilayer	ABCO‐bilayer
#^a)^	20	24	19	20	20	30
I (nA)^b)^	109.3	74.1	59.1	31.6	19.1	68.7
σ (nA)^c)^	14.2	20.0	16.7	11.0	6.0	27.3

^a)^ The number of measurements in 2–3 different samples.

^b)^ Mean value of current at + 0.5 V.

^c)^ Standard deviation value of current at +0.5 V.

(3) $$I_{0}=I_{0}e^{-\beta d}$$

where *β* is the tunneling attenuation factor, *d* is the width of the energy barrier (often assumed to be similar to the molecular length), and *I*_0_ is the extrapolated current when *d* = 0. According to the Simmons model, tunneling current decreases exponentially as the thickness of tunneling barrier increases, exhibiting a positive value of attenuation factor. The ZnOEP system followed this trend in both STS and EGaIn junction measurements, showing higher tunneling currents for the monolayer than the ABCO‐bilayer (Figure 3a,b). The currents of the DABCO‐bilayer were indistinguishable from those of the ABCO‐bilayer in STS, considering the error ranges. EGaIn junction data corroborated this finding (Figure 3b).

In contrast, the PdOEP system exhibited a markedly different trend. Despite the monolayer being thinner than the ABCO‐bilayer, the bilayer exhibited higher currents than the monolayer. The DABCO‐bilayer, with a thickness nearly identical to the ABCO‐bilayer, showed lower current and current density values than the monolayer while the ABCO‐bilayer exhibited substantially higher current and current density than the DABCO‐bilayer (Figure 3c,d). The higher values in thicker molecular layers suggest a negative attenuation behavior, indicating potential for highly conductive molecular wires with long‐range charge transport.^[^ ^26^, ^27^ ^]^

Negative tunneling attenuation behaviors have been previously observed in molecular wires composed of π‐extended building blocks such as thiophene,^[^ ^28^ ^]^ diketopyrrolopyrrole,^[^ ^29^ ^]^ and porphyrin^[^ ^30^ ^]^ units. Widely accepted reasons to explain the unusual behaviors include i) stronger internal coupling of molecule than molecule‐electrode coupling as the molecular length increases, ii) compression of HOMO‐LUMO gaps with increasing molecular length, and iii) stable radical structure leading to strong delocalization.^[^ ^26^, ^27^ ^]^ Our approach is distinct from the prior studies in that we modulate supramolecular molecular orbital distribution through deliberate variations in metal–ligand interactions. The negative attenuation behavior in the PdOEP system was further supported by conductiveprobe‐AFM imaging. Figure 3e shows the coexistence phase of the PdOEP monolayer and ABCO‐based bilayer. The 3D topographic image confirmed a depth difference of ∼5 Å between bilayer and monolayer. The current through the bilayer (12.28 ± 0.02 nA) was ≈1.2 times higher than that through the monolayer (10.63 ± 0.05 nA) (Figure 3f).

### 2.4 Calculations

**Figure 4:**
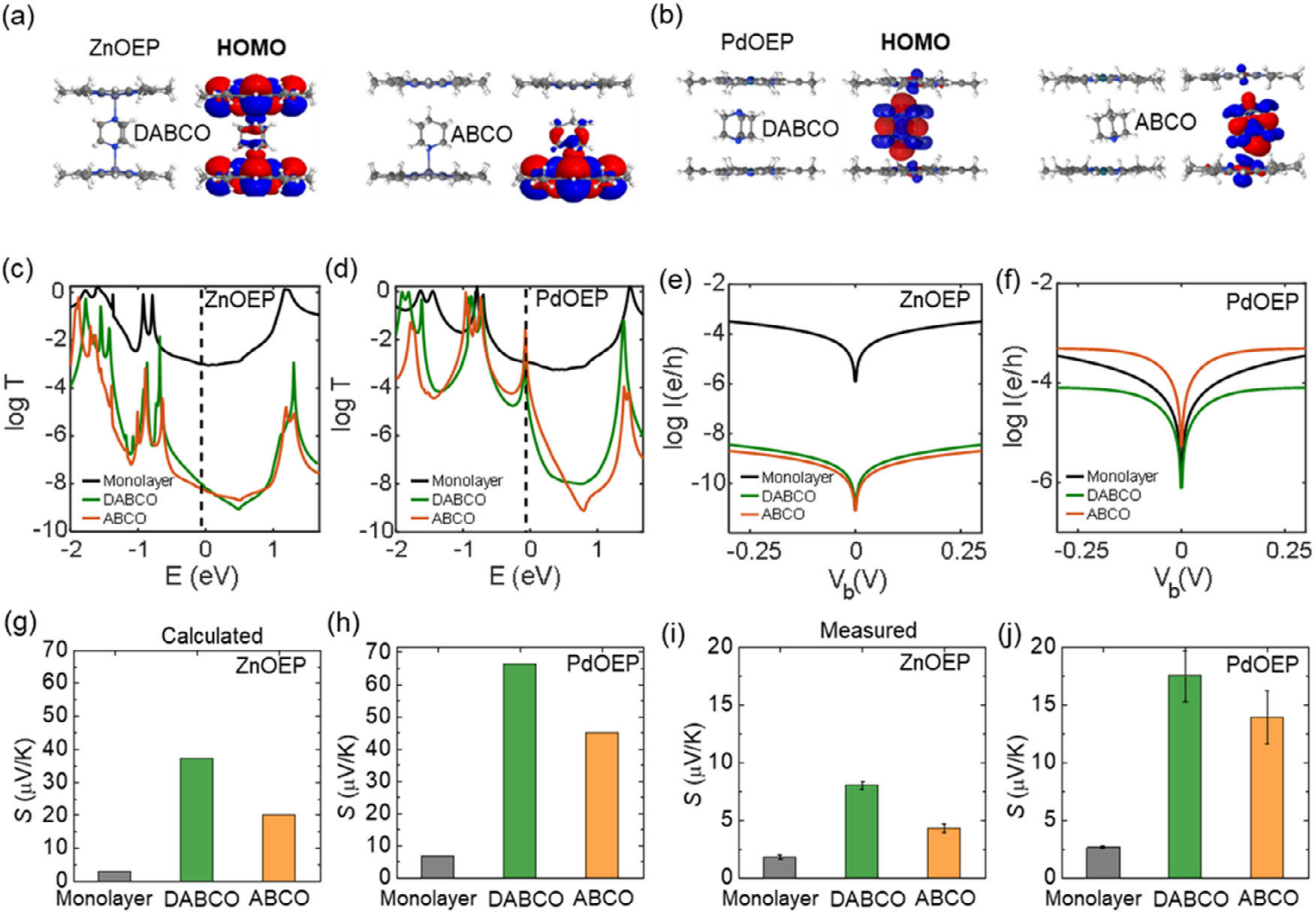
a,b) The ground state structure of ZnOEP and PdOEP bilayers with two different molecular spacers (ABCO and DABCO). Density functional theory (DFT) transmission coefficient for monolayer, DABCO‐bilayer and ABCO‐bilayer with c) Zn and d) Pd metal site. Current–voltage (*I–V*) relationship for monolayer, DABCO‐bilayer and ABCO‐bilayer with e) Zn and f) Pd metal site. In c and f, e is the electron charge and h is the Planck's constant. g,h) Calculated room‐temperature Seebeck coefficient for monolayer, DABCO‐bilayer and ABCO‐bilayer of ZnOEP and PdOEP. i,j) correspondent measured Seebeck coefficient for bilayers.

To investigate the charge‐transport properties of monolayer, DABCO‐bilayer, and ABCO‐bilayer (**Figure** **4**a,b) between electrodes, we first calculated the ground‐state geometries and electronic structures of the gas‐phase molecules using the SIESTA^[^ ^31^ ^]^ implementation of DFT, as detailed in the Supporting Information. Our findings indicate that the ground‐state geometry of ZnOEP‐DABCO and ZnOEP‐ABCO bilayers is significantly influenced by the Zn(II) metal site, which forms strong coordination bonds with the nitrogen atoms of the pillar molecules DABCO and ABCO (Figures S11, S13, Supporting Information). In contrast, the Pd(II) metal site forms weaker interactions with nitrogen atoms of the pillar molecules, establishing ion‐induced dipole interaction primarily with the hydrogen atoms in ABCO (Figure S12, Supporting Information). The binding energy calculations also support this (see Figure S18, Supporting Information).

The interaction between the ZnOEP and the pillar molecule leads to variations in charge distribution across the molecular orbital, as illustrated in Figure 4a. In ZnOEP‐DABCO, the charge distribution is mainly localized on porphyrins (Figure 4a; Table S1, Supporting Information), whereas in PdOEP‐ABCO, the state is hybridized, localized on one of the porphyrins and partly on the pillar molecule (Figure 4b; Table S2, Supporting Information). This disparity is reflected in the electrical properties of the junctions as follows. To understand the electrical and thermoelectric properties, we formed junctions with different molecules between graphene electrodes (see the junction structures including molecules between electrodes in Figures S14–S16, Supporting Information). We then calculated the ground state geometry and mean field Hamiltonians of the structures, including the electrodes, and used scattering theory^[^ ^32^ ^]^ to calculate transmission probability *T*(*E*) of electrons with energy *E* from one electrode to the other using GOLLUM transport code.^[^ ^33^ ^]^ Figure 4c,d show the *T*(*E*) for junctions formed by Zn and Pd porphyrins, respectively. The transmission amplitude (and consequently room‐temperature electrical conductance as shown in Figure S14, Supporting Information) near the Fermi energy for the ZnOEP‐based assemblies ranks as follows: monolayer > DABCO‐bilayer > ABCO‐bilayer. We then calculated zero bias current–voltage (*I–V*) characteristics of the junctions using Landauer formula (see the Supporting Information for details). The calculated *I–V* characteristics for the monolayer, DABCO‐bilayer, and ABCO‐bilayer followed the same conductance order, as depicted in Figure 4e, aligning with our experimental results.

In contrast, changing the metal center from Zn(II) to Pd(II) significantly alters the electronic properties. Calculations showed that the interaction of PdOEP with the pillar molecules is weaker than that of ZnOEP. PdOEP does not establish strong coordination bonds between Pd(II) and the pillar molecules, while Pd(II) forms weaker coordination bonds with the nitrogen atoms and stronger interactions with the hydrogen atoms (Figure S12, Supporting Information). The molecular orbitals depicted in Figure 4b and Table S2 (Supporting Information) reveal that the charge distribution is primarily localized on the central part of the molecule (i.e., pillar molecules) in both ABCO and DABCO. As a result, a new state (due to the HOMO of pillar molecules – see also local density of state calculation in Figure S16, Supporting Information) forms between the state of porphyrin and near the DFT Fermi energy, leading to a smaller HOMO‐LUMO gap (Figure 4d; Figure S17, Supporting Information). Note that, despite reduced HOMO‐LUMO gap of PdOEP bilayers, the binding energy calculations (Figure S18, Supporting Information) confirm that PdOEP bilayers exhibits a weaker binding affinity with pillar molecules in the gas phase compared to ZnOEP bilayers. Consequently, the *T*(*E*) magnitude (and room‐temperature electrical conductance as shown in Figure S14, Supporting Information) for the PdOEP‐based assemblies followed the order of ABCO‐bilayer > DABCO‐bilayer > monolayer. The calculated *I–V* characteristics for the monolayer, DABCO‐bilayer, and ABCO‐bilayer indicate the same order, as shown in Figure 4f, which is in agreement with our experimental results.

### 2.5 Thermoelectric Properties

Given that the gradient of ln(*T*(*E*)) at *E*_F_ corresponds to the Seebeck coefficient (*S*, µV K^−1^) of the junction, as shown in the above Equation (2), our DFT calculation results can be further validated by thermopower measurements. The calculated transmission plots indicated that the gradient of ln(*T*(*E*)) at *E*_F_ for ZnOEP assemblies decreases in the order of DABCO‐bilayer > ABCO‐bilayer > monolayer (Figure 4g). This trend should mirror the *S* values (Figure 4i) (see the Supporting Information for details about theoretical estimation of *S* values). We conducted thermopower measurements using the EGaIn technique following the previously reported procedures (Figures S21, S22, Supporting Information).^[^ ^34^ ^]^ In ZnOEP assemblies, the *S* values were measured to be 2.6 ± 0.1, 13.9 ± 2.3, 17.4 ± 2.4, and µV K^−1^ for the monolayer, ABCO‐bilayer, and DABCO‐bilayer, respectively. This trend is qualitatively in agreement with the expected trend (Figure 4i). Similarly, the trend of calculated *S* values for PdOEP assemblies (Figure 4h) aligned with the trend of experimentally measured *S* values (Figure 4j): monolayer (4.3 ± 0.4 µV K^−1^) < ABCO‐bilayer (4.3 ± 0.4 µV K^−1^) < DABCO‐bilayer (8.0 ± 0.3 µV/K). The PdOEP bilayers exhibited higher *S* values than the ZnOEP bilayers, reflecting the effect of weak metal‐pillar ligand interactions. The *S* values were higher in calculations compared to measurements, which may be due to the inhomogeneity of monolayers in experiments compared to the ideal junctions in the calculations.^[^ ^35^ ^]^

## 3 Conclusion

This work demonstrates the profound impact that significant changes in metal‐ligand interactions can have on charge transport properties of supramolecular assemblies, particularly those composed of metalloporphyrins and axial pillar ligands as a proof‐of‐concept. The PdOEP and ZnOEP systems exhibit distinct electrical behaviors due to nuanced differences in their coordination bonding strengths with pillar ligands. ZnOEP forms stronger coordination bonds, leading to conventional tunneling attenuation and low thermopower. In contrast, PdOEP, with its weaker interactions, shows an atypical negative attenuation behavior and high thermopower. These findings are attributed to the significant variations in metal–ligand interactions that affect the molecular orbital distribution within the supramolecular assemblies. Quantum‐chemical calculations indicate that the strong metal‐pillar ligand interactions in ZnOEP localizes frontier molecular orbitals on the porphyrin units, while the weak interactions in PdOEP isolate frontier molecular orbitals on the pillar molecule, compressing the HOMO‐LUMO gap and enhancing electrical conductance in thicker bilayers. Our results highlight the importance of tuning coordination strength in metal–ligand systems to control charge transport behavior. While strong interactions (e.g., ZnOEP‐ligand) offer structural robustness, weaker coordination (e.g., PdOEP‐ligand) can enhance tunneling efficiency and thermoelectric response. These insights provide a useful guideline: selecting metal centers and ligands to balance binding strength and electronic coupling is key to optimizing performance in molecular electronic devices.

## Conflict of Interest

The authors declare no conflict of interest.

## Supporting information



Supporting Information

## Data Availability

The data that support the findings of this study are available in the supplementary material of this article.
