# Supplementary material for: Engineering Charge Transport by Tunneling in Supramolecular Assemblies through Precise Control of Metal–Ligand Interactions
Source: Small. 2025 May 23;21(27):2501303. doi: 10.1002/smll.202501303 (PMC12243708; doi:10.1002/smll.202501303)
Supplement: Supplementary file 1 — Supporting Information [file SMLL-21-2501303-s001.docx]

Supporting Information
©Wiley-VCH 2025
69451 Weinheim, Germany

Engineering Charge Transport by Tunneling in Supramolecular Assemblies Through Precise Control of Metal-Ligand Interactions

Hungu Kang,^[a]†^ Abdalghani H. S. Daaoub,^[b]†^ Sara Sangtarash,^[b]^ Jiung Jang,^[a]^ Kangsik Lee,^[a]^ Hatef Sadeghi*^[b]^ and Hyo Jae Yoon*^[a]^

^†These authors contributed equally to this work.^

**Abstract:** Coordination-driven supramolecular assemblies are promising for nanometer-sized electronic devices due to the potential to manipulate metal-ligand interactions and thereby control charge transport via tunneling through these assemblies. We investigate cross-plane charge tunneling in assemblies of metalloporphyrins and pillar molecules, specifically palladium(II) and zinc(II) octaethylporphyrin (PdOEP and ZnOEP) monolayers and bilayers with bidentate (DABCO) and monodentate (ABCO) pillar ligands on highly oriented pyrolytic graphite (HOPG). Junction measurements and quantum-chemical calculations reveal that metal-ligand interactions significantly influence charge transport via tunneling and thermoelectric effects. Weak interactions in PdOEP assemblies create isolated molecular orbitals on interior pillar ligands, compressing the HOMO-LUMO gap and enhancing tunneling currents with unusual, inverted attenuation behavior and high thermopower. Conversely, strong interactions in ZnOEP assemblies induce localized orbitals on the porphyrin, leading to conventional tunneling decay behavior and low thermopower. Our study highlights the potential of metal-ligand interactions as a strategy to engineer molecular orbital distribution, enhancing quantum transport efficiency in molecular-scale devices.

DOI: 10.1002/anie.2025XXXXX

Table of Contents

1. Experimental Details………………………………………………………………………………………………………………………………………………………….……2

2. Electrical Measurements…………………………………………………………………………………………………………………………………………………….…….2

3. Characterization…………………………………………………………………………………………………………………………………………………………………….2

4. Computational Methods ……………………………………………………………………………………………………………………………………………………………3

5. Supporting Figures …………………………………………………………………………………………………………………………………………………………………4

6. References ………………………………………………………………………………………………………………………………………………………………….……. 18

Experimental Procedures

**1. Experimental Details**

**1.1 Molecules and Materials**

All reagents were used as supplied unless otherwise specified. All organic solvents were purchased from Sigma-Aldrich. PdOEP and ZnOEP (purity ≥ 85 %), DABCO (purity ≥ 97 %), and ABCO (purity ≥ 97 %) were purchased from Sigma-Aldrich. Highly ordered pyrolytic graphite (HOPG; 12 mm $\times$12 mm $\times$ 2mm; ZYB grade) was purchased from BRUKER. High purity EGaIn (99.99%) was obtained from Sigma-Aldrich and used as supplied. ^1^H and ^13^C NMR spectra were recorded on a Bruker FT-NMR Advance-500 using CDCl_3_ as a solvent and residual solvents as an internal standard. Chemical shifts are expressed in parts per million (ppm) relative to internal TMS and coupling constants (*J*) are in Hertz.

**1.2. Preparation of Porphyrin Bilayer on Substrate**

We prepared SAMs following a previously reported procedure.^[1]^ First, a freshly prepared HOPG substrate was immersed in a saturated solution of porphyrin (ZnOEP or PdOEP) in tetradecane (~3 mM). After 5 minutes of incubation at room temperature, the HOPG//porphyrin chip was removed from the solution and gently rinsed with pure tetradecane (3 × 1 mL). The HOPG//porphyrin chip was then immersed in a 3 mM solution of pillar molecules (ABCO or DABCO) in tetradecane. After 15 minutes of incubation at room temperature, the HOPG//porphyrin/pillar chip was removed from the solution and rinsed with pure tetradecane (3 × 1 mL). The HOPG//porphyrin/pillar chip was again immersed in a saturated solution of porphyrin in tetradecane to fabricate the porphyrin bilayer. After 5 minutes of incubation at room temperature, the HOPG//porphyrin/pillar/porphyrin chip was removed from the solution and rinsed with pure tetradecane (3 × 1 mL). Finally, the solvent on the porphyrin bilayer was then evaporated with an N_2_ gun for a few seconds.

**1.3. UV/Vis and ^1^H NMR spectroscopy**

Titration experiments were performed using UV/Vis and ^1^H NMR spectroscopy with a fixed concentration of ZnOEP and PdOEP (1 × 10^-5^ M) while varying the molar ratios of DABCO. The association constant (*K*_a_) for a 1:1 equilibrium is determined using the following equation (1):

$K_{a}= \frac{[HG]}{\left[ H \right][G]}$ (1)

Here, [H], [G], and [HG] represent the concentrations of the host, guest, and complex, respectively. In this study, the porphyrin serves as the host and the ligand as the guest. The host concentraiton is kept constant while varying the guest concentration during titration. The *K*_a_ is obtained through global fitting of the titration data using a 1:1 binding model, implemented via the web applet at supramolecular.org web.^[2]^ In the ^1^H NMR spectroscopic experiments, a 50 mM CDCl_3_ solution of DABCO was incrementally added to a 10 mM solution of ZnOEP and PdOEP, and the resulting changes in proton chemical shift were monitored to observe the binding interactions.

Solid-state UV-vis measurements were performed on PdOEP and ZnOEP monolayers and bilayers assembled on single-layer graphene/glass substrates. Bilayers were formed using DABCO and ABCO as pillar molecules. The higher absorbance observed in the bilayer samples indicates increased porphyrin loading relative to the monolayers.

**2. Electrical Measurements**

**2.1 EGaIn Technique**

Junction formation and measurements for current density and Seebeck coefficient were performed following methods reported in the literature.^[3]^ For thermopower measurements, we varied the temperature of the hot chuck from 290 to 310 K. To derive the Seebeck coefficient (*S*, *μ*V/K) value, histograms of thermovoltage (*∆V*, *μ*V) at different temperature differentials (∆*T* = 4, 8, and 12 K) were obtained. From the histograms, Gaussian-based mean and standard deviation of ∆*V* were extracted.

**2.2 STS Measurements**

STS measurements in the ambient conditions were performed following a method reported in the literature.^[1]^ For the *I-V* recording step, the following sub-steps are involved. 1) A molecularly resolved STM image should be obtained, and then the tip is immediately positioned on the target molecular spot. 2) The current-voltage curve is recorded from +1.0 V to –1.0 V at 10 Hz while the electrode separation is fixed. 3) Upon completion of the *I-V* curve, the tip is pulled away from the substrate until the current drops to a preset value, and the data collection process is repeated.

**3. Characterization**

**3.1 Surface Characterization with XPS**

The XPS measurements were carried out on a Thetaprobe (Thermo) with a monochromated Al Kα (1486.6 eV) source. The measurements were done at room temperature in a vacuum of 6.7 × 10^-8^ Pa. The N1s XPS spectra of the porphyrin layers on HOPG were examined to obtain information regarding the chemical structure and composition of the porphyrins. Each peak positions and shapes of the core level photoelectron spectra were analyzed with a XPS Peak Fit program, in which a Shirley-type background correction was utilized.

**3.2 Surface Characterization with AFM and STM**

Large area AFM and high-resolution STM were used to study the structure of metalloporphyrin monolayers and bilayers. AFM measurements were conducted in non-contact mode of Multimode (Bruker, Santa Barbara, CA). To calculate the surface area of regions with similar height, we used NanoScope Analysis software (Bruker). A height-based AFM image of the sample was first obtained, and the software processed the pixel height data to generate a histogram. Based on this, a threshold height was defined to segment the image. Conneted pixel regions above the threshold were highlighted and quantified, enabling selective analysis of areas with distinct height features. This method allowed us to monitor changes in surface coverage as a function of porphyrin deposition time, reflecting the kinetics of surpamolecular assembly.

STM measurements were performed using a Nanoscope V (Bruker) with a commercial Pt/Ir (80/20) tip. All STM images were obtained using a constant height and current mode under ambient conditions at room temperature. Imaging conditions were in the range of -1100 to -1200 mV for the bias voltages and in the range of 0.30 to 0.50 nA for tunneling currents between the tip and sample. The AFM and STM images of the metalloporphyrin (ZnOEP and PdOEP) layers are shown in Figure S3-S9.

**3.3 CP-AFM Measurements**

CP-AFM images were obtained by a Bruker AFM Multimode with TUNA Application Module (Bruker). TUNA (tunneling AFM) is designed for ultra-low current measurement on low-conductivity samples. An Au-coated silicon nitride tip with a nominal radius of 130 nm (NPG-10, Bruker; resonant frequency = 23 kHz, spring constant = 0.12 N/m) was used to contact the porphyrin layers in TUNA mode. The calculated average value for the set-point force was 8.9 nN for all samples during CP-AFM measurements. Both topography and tunneling current images were obtained simultaneously.

**4. Computational Methods**

The optimized geometry and ground state Hamiltonian and overlap matrix elements of each structure (as shown in Figure 4a,b) was self-consistently obtained using the SIESTA implementation^[4]^ of density functional theory (DFT). SIESTA employs norm-conserving pseudo-potentials to account for the core electrons and linear combinations of atomic orbitals to construct the valence states. The generalized gradient approximation (GGA) of the exchange and correlation functional is used with the Perdew-Burke-Ernzerhof parameterization (ca) a double-ζ polarized (DZ) basis set, a real-space grid defined with an equivalent energy cut-off of 150 Ry. The geometry optimization for each structure is performed to the forces smaller than 10 meV/Å. The mean-field Hamiltonian obtained from the converged DFT calculation was combined with Gollum^[5]^ implementation of the non-equilibrium Green’s function method**^3^** to calculate the phase-coherent, elastic scattering properties of the each system consist of left gold (source) and right gold (drain) leads and the scattering region. The transmission coefficient *T(E)* for electrons of energy *E* (passing from the source to the drain) is calculated via the relation: $T\left( E \right)=Trace\left( \Gamma_{R}(E)G^{R}(E)\Gamma_{L}(E)G^{R\dagger}(E) \right)$. In this expression,$\Gamma_{L,R}\left( E \right)=i\left( \sum_{L,R}\left( E \right)-{\sum_{L,R}}^{\dagger}\left( E \right) \right)$ describe the level broadening due to the coupling between left (L) and right (R) electrodes and the central scattering region, $\sum_{L,R}\left( E \right)$are the retarded self-energies associated with this coupling and $G^{R}=\left( ES-H-\sum_{L}-\sum_{R} \right)^{-1}$ is the retarded Green’s function, The DFT+∑ approach has been employed for spectral adjustment.^3^ The room temperature current voltage characteristic is then calculated using the Landauer formula $I(V,T)=e/h\int_{-\infty}^{+\infty} dE T\left( E \right)\left( f\left( E,T,{-V}_{b}/2 \right)-f(E,T,V_{b}/2) \right)$, where $f={(e^{(E-E_{F})/k_{B}T}+1)}^{-1}$ is the Fermi-Dirac probability distribution function, *T* is the temperature, *E_F_* is the Fermi energy, *e* is electron charge and *h* is the Planck’s constant. The Seebeck coefficient is given by $S\left( E_{F},T \right)=-\frac{L_{1}}{eTL_{0}}$ where $L_{n}=\int_{-\infty}^{+\infty} dE T\left( E \right)\left( -df\left( E,T{,E}_{F} \right)/dE) \right)$. To calculate Ionization Potential (IP) and the Electron Affinity (EA) which can give a more accurate estimation of the HOMO and LUMO energy gap. $IP=E\left( N-1 \right)-E\left( N \right),EA=E\left( N \right)-E\left( N+1 \right),$ where$E(N)$ is the ground state energy of the neutral molecule.$E\left( N-1 \right)$ is the ground state energy with one electron removed.$E(N+1)$ is the ground state energy with one electron added.

Supporting Figures


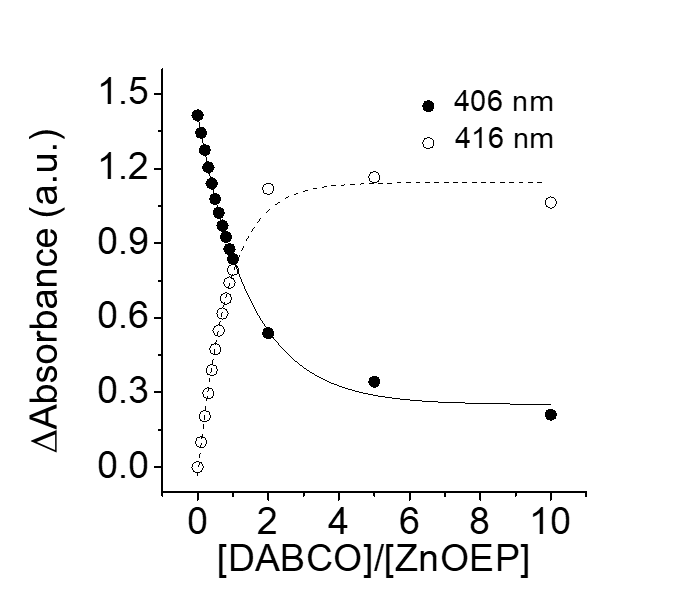


**Figure S1.** Absorbance variation of ZnOEP at 416 nm (white circles) and 406 nm (black circles) with different DABCO molar ratios.


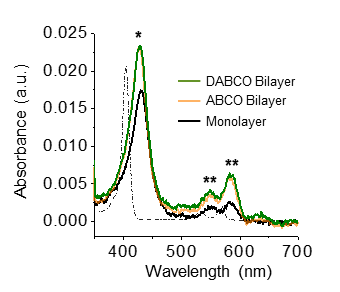


**Figure S2.** UV-vis absorption spectra of ZnOEP monolayer and bilayers assembled with different pillar molecules (DABCO and ABCO) on single-layer graphene. The dotted line represents the spectrum of ZnOEP in solution as a reference. Single (*) and double (**) asterisks denote the B band (380-500 nm) and the Q band (500-700 nm) transitions, respectively.


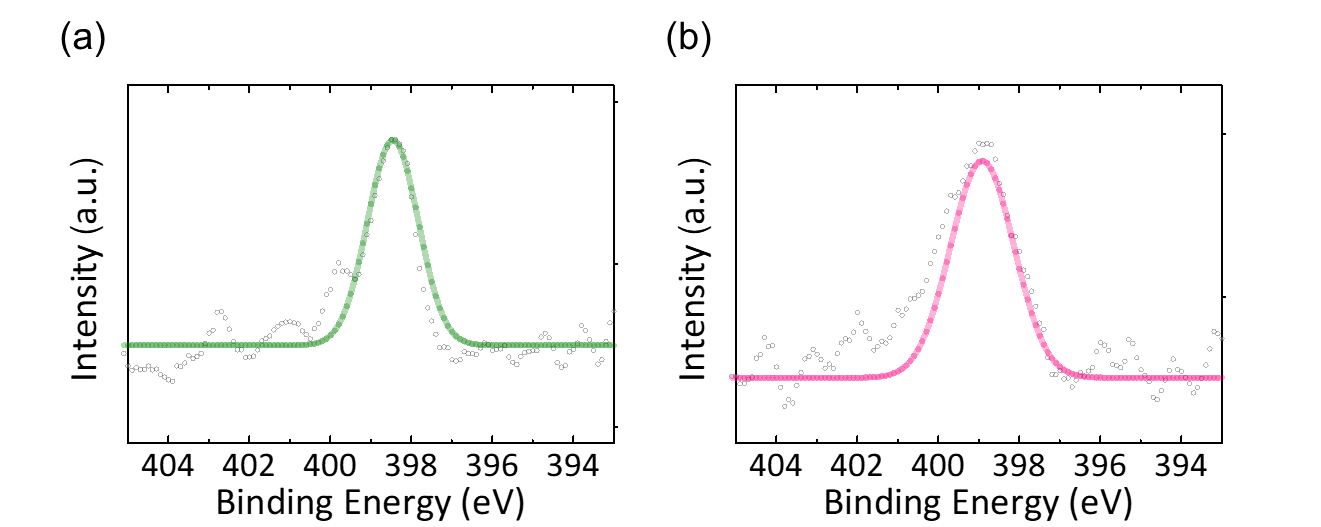


**Figure S3.** High-resolution X-ray photoelectron spectra of the nitrogen (N1s) core-level between (a) ZnOEP and (b) PdOEP monolayer structures.


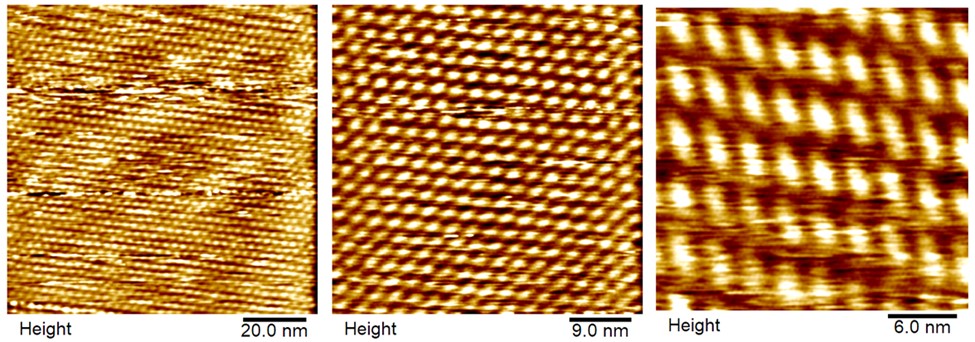


**Figure S4.** The STM images of ZnOEP monolayer on HOPG.

**
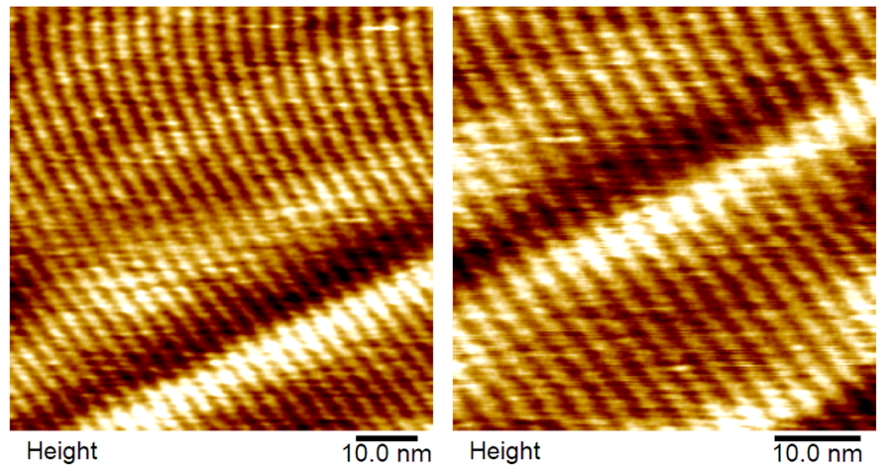
**

**Figure S5.** The STM images of ZnOEP-ABCO bilayer on HOPG.

**
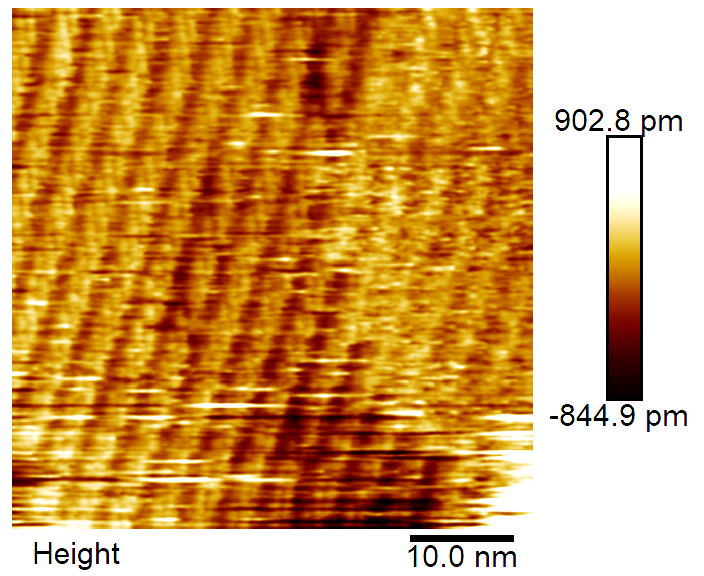
**

**Figure S6.** The STM images of ZnOEP-DABCO bilayer on HOPG.


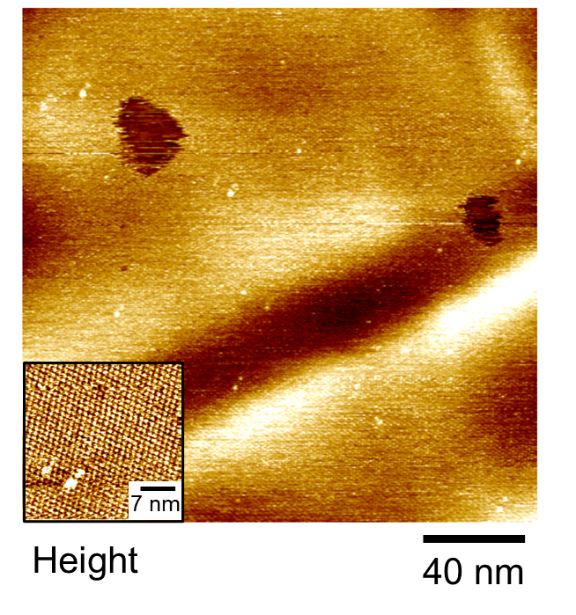
**Figure S7.** Large area STM image of PdOEP-DABCO bilayer (200 × 200 nm^2^) on HOPG.

**
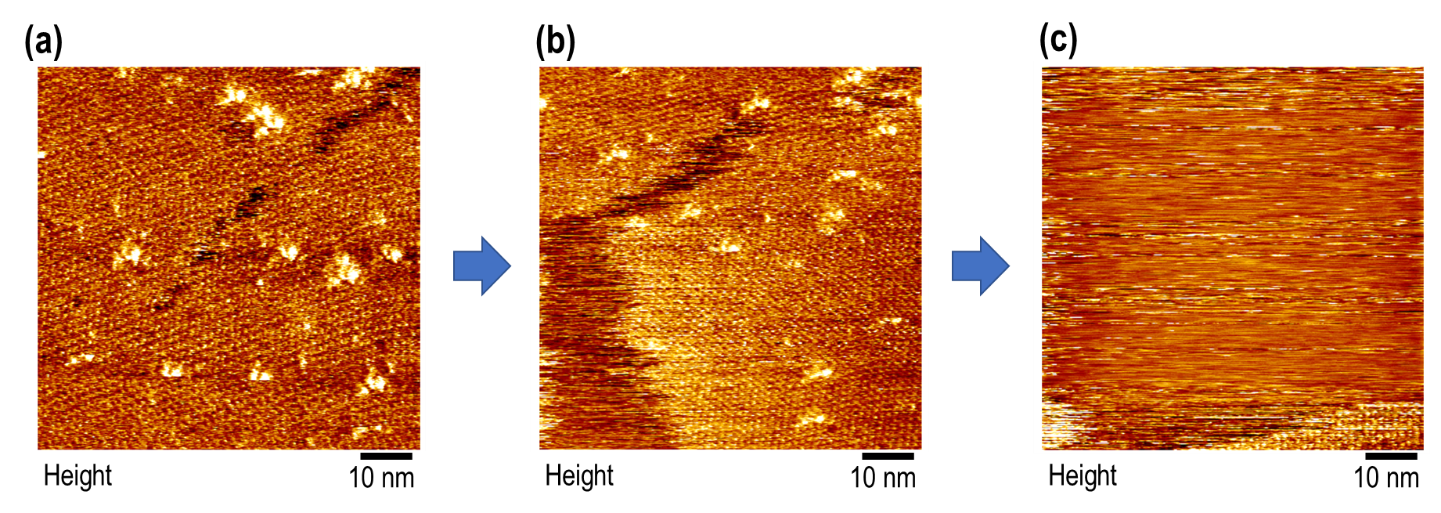
**

**Figure S8.** The STM images of PdOEP-ABCO bilayer after (a) first, (b) second, and (c) third scanning in same area.


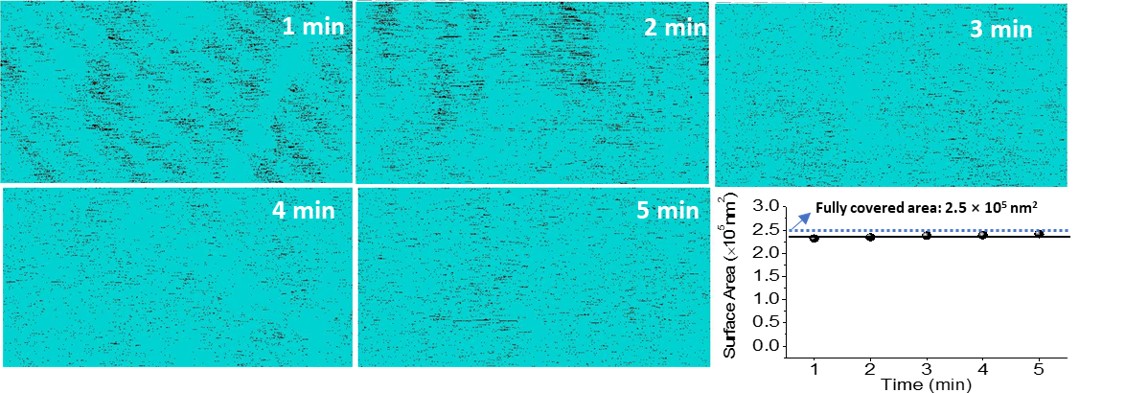


**Figure S9.** The AFM images showing the changes in surface coverage of the ZnOEP_outer_ (cyan color) on the ZnOEP_inner_-ABCO structure (brown color) as a function of the ZnOEPouter deposition time (from 1 to 5 min). And the variation in the surface area of the ZnOEP_outer_ layers as a function of the deposition time of the ZnOEP_outer_ layer


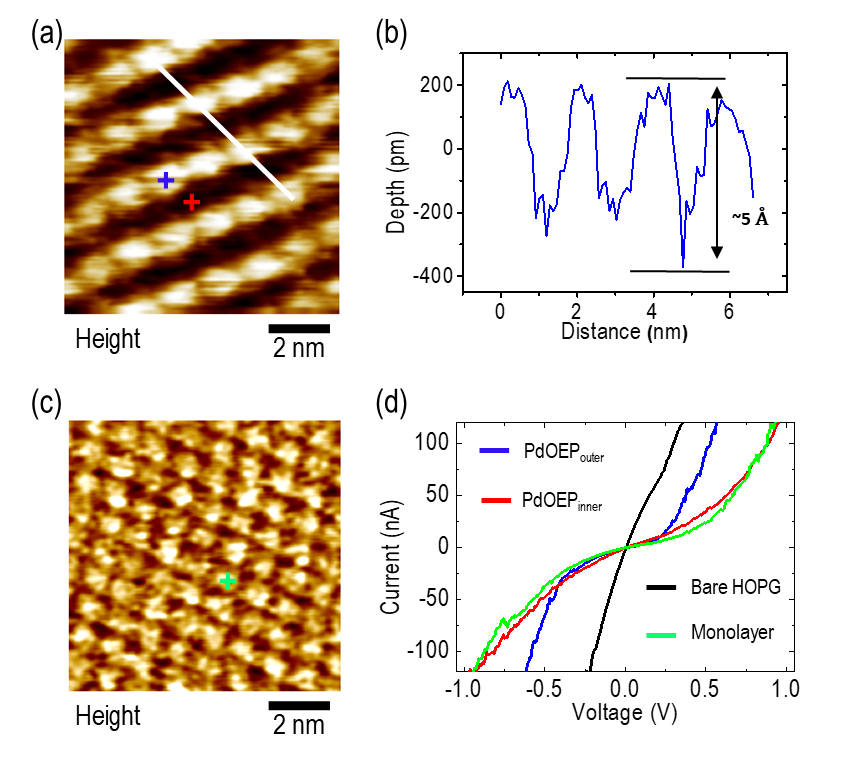
**Figure S10.** (a) STM image and (b) corresponding height profile of the β phase in the PdOEP-ABCO bilayer on HOPG, showing a ~5 Å step height consistent with the distance between PdOEP_inner_ and PdOEP_outer_ layers. (c) STM image of a PdOEP monolayer on HOPG. (d) Selective I-V measurements from STM images of (a) and (c): red and blue curves correspond to the PdOEP_inner_ and the PdOEP_outer_ regions in (a), respectively; green curve is from the PdOEP monolayer in (c); and black curve is from bare HOPG. The similarity between the red and green curves confirms that the dark region in (a) corresponds to the PdOEP_inner_ structure, not bare HOPG.

**Figure S11.** Total energy differences for range of configurations with different position of different molecular spacers (ABCO vs. DABCO) between the structure of ZnOEP bilayers. See Figure S12 for details of different position of DABCO between the structure of ZnOEP bilayers.

**Figure S12.** Total energy differences for range of configurations with different position of different molecular spacers (ABCO vs. DABCO) between the structure of PdOEP bilayers.

**Figure S13.** Range of configurations with different position of different molecular spacers DABCO between the structure of ZnOEP bilayers.

**Table S1. F**rontier orbitals of relaxed structure of monolayer, DABCO-bilayer and ABCO-bilayer for ZnOEP molecules from HOMO-4 to LUMO+4 orbitals.

**Table S2.** Frontier orbitals of relaxed structure of monolayer, DABCO-bilayer and ABCO-bilayer for PdOEP molecules from HOMO-4 to LUMO+4 orbitals.

**Figure S14.** **Transmission and electrical conductance properties. (**a) Structure of sandwiched molecular junctions with monolayer, DABCO-bilayer and ABCO-bilayer of PdOEP molecules between two graphene electrodes. (b,d) Transmission coefficient and (c,e) electrical conductance for monolayer, DABCO-bilayer and ABCO-bilayer of ZnOEP and PdOEP molecules, respectively.

$$e^{-}$$

$$e^{-}$$

Top Lead

Bottom Lead

Top Lead

Bottom Lead

Bottom Lead

Top Lead

**Figure S15.** Local density of state around resonance (HOMO and LUMO resonance in transmission plots) for monolayer, DABCO-bilayer and ABCO-bilayer of ZnOEP molecules.

$$e^{-}$$

Right Lead

Left Lead

Left Lead

Right Lead

Left Lead

Right Lead

DABCO

Right Lead

Left Lead

ABCO

ABCO

Right Lead

Left Lead

Right Lead

Left Lead

DABCO


$$e^{-}$$

Left Lead

Right Lead

Left Lead

Right Lead

Right Lead

Left Lead

ABCO

DABCO

Right Lead

Left Lead

ABCO

Right Lead

Left Lead

DABCO

Right Lead

Left Lead

**Figure S16.** Local density of state around resonance (HOMO and LUMO resonance in transmission plots) for monolayer, DABCO-bilayer and ABCO-bilayer of PdOEP molecules.

**Figure S17.** (a,c) calculated HOMO amd LUMO energy gab for monolayer, DABCO-bilayer and ABCO-bilayer of PdOEP and ZnOEP molecules. (b,d) Ionization potential (IP) and the electron affinity (EA) for monolayer, DABCO-bilayer and ABCO-bilayer of PdOEP and ZnOEP molecules.


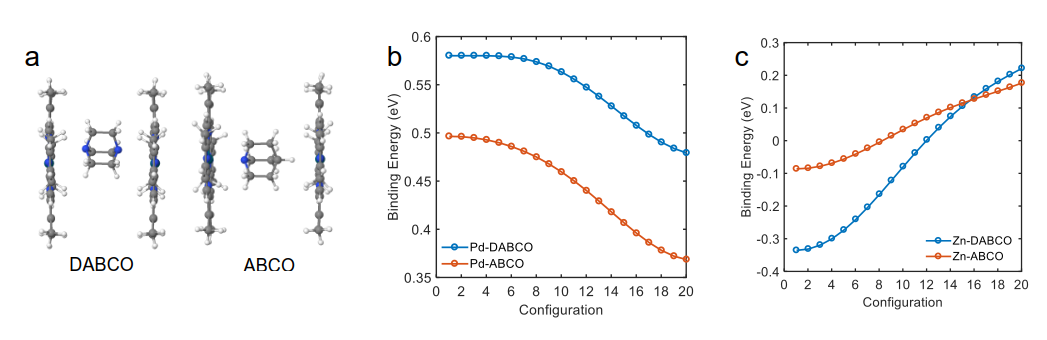


**Figure S18.** (a) The ground state structure of PdOEP bilayer with two different molecular spacers (ABCO and DABCO). (b) binding energy between monolayer, DABCO-bilayer and ABCO-bilayer of PdOEP molecule, (c) binding energy between monolayer, DABCO-bilayer and ABCO-bilayer of ZnOEP molecule.

**Figure S19.** Charge transfer between the porphyrins and the encapsulated molecule (denoted by C) for PdOEP where $\Delta Q_{x}=Q_{x}^{i}-Q_{x}^{b}$, where $Q_{x}^{i}$ is charge of isolated C, and $Q_{x}^{b}$ is charge of C between two metalloporphyrin (PdOEP). This is based on Mulliken charge analysis, obtained from DFT calculations, of isolated spacer molecules and spacer-bridged pillar molecule bilayers.

**Figure S20.** Charge transfer between the porphyrins and the encapsulated molecule (denoted by C) for ZnOEP where $\Delta Q_{x}=Q_{x}^{i}-Q_{x}^{b}$, where $Q_{x}^{i}$ is charge of isolated C, and $Q_{x}^{b}$ is charge of C between two metalloporphyrin (ZnOEP). This is based on Mulliken charge analysis, obtained from DFT calculations, of isolated spacer molecules and spacer-bridged pillar molecule bilayers.

**
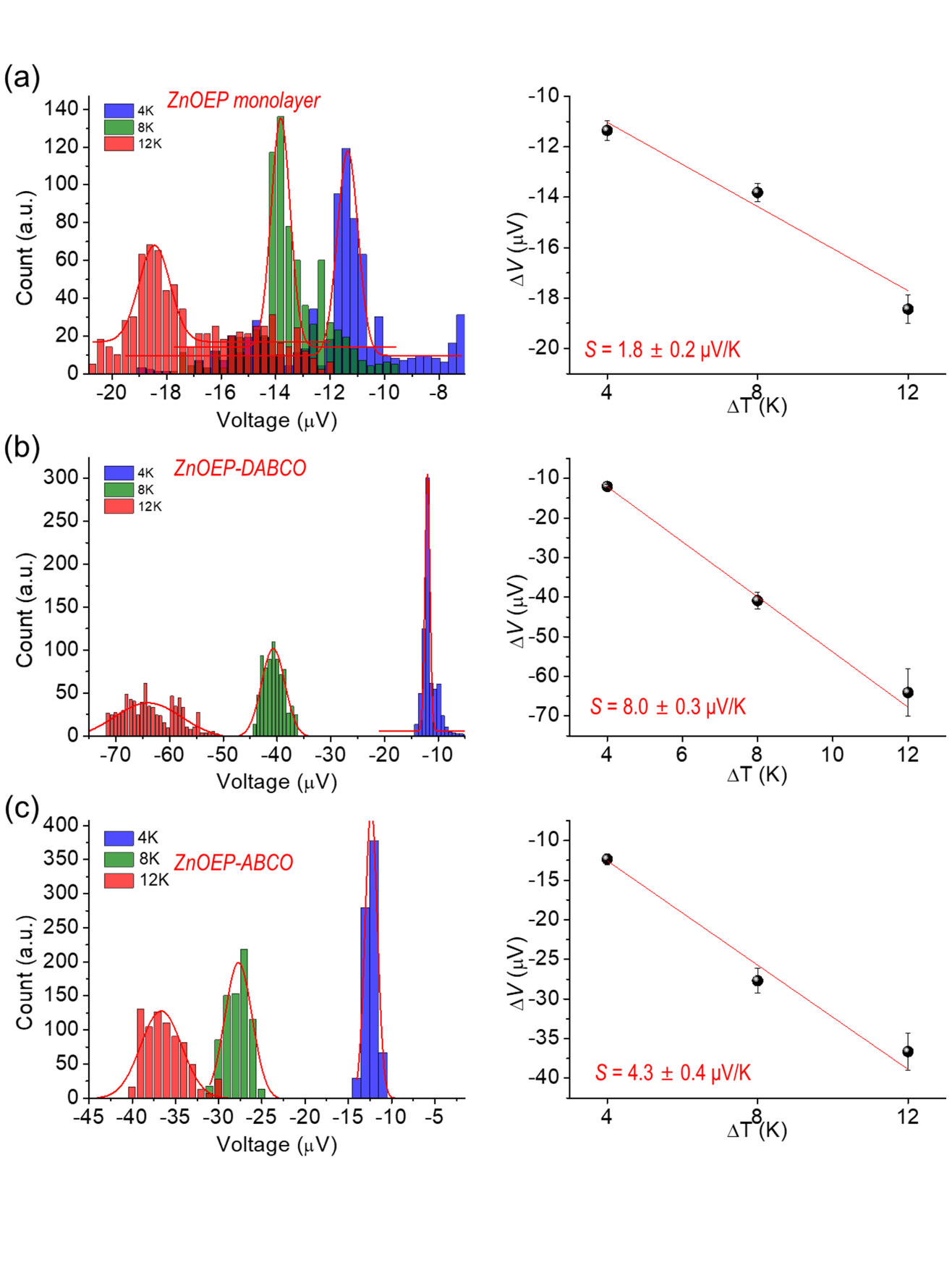
Figure S21.** Histograms of thermovoltage (Δ*V*, μV) and plots of Δ*V* as a function of Δ*T* for (a) ZnOEP monolayer, (b) ZnOEP-DABCO bilayer, and (c) ZnOEP-ABCO bilayer junctions.

**
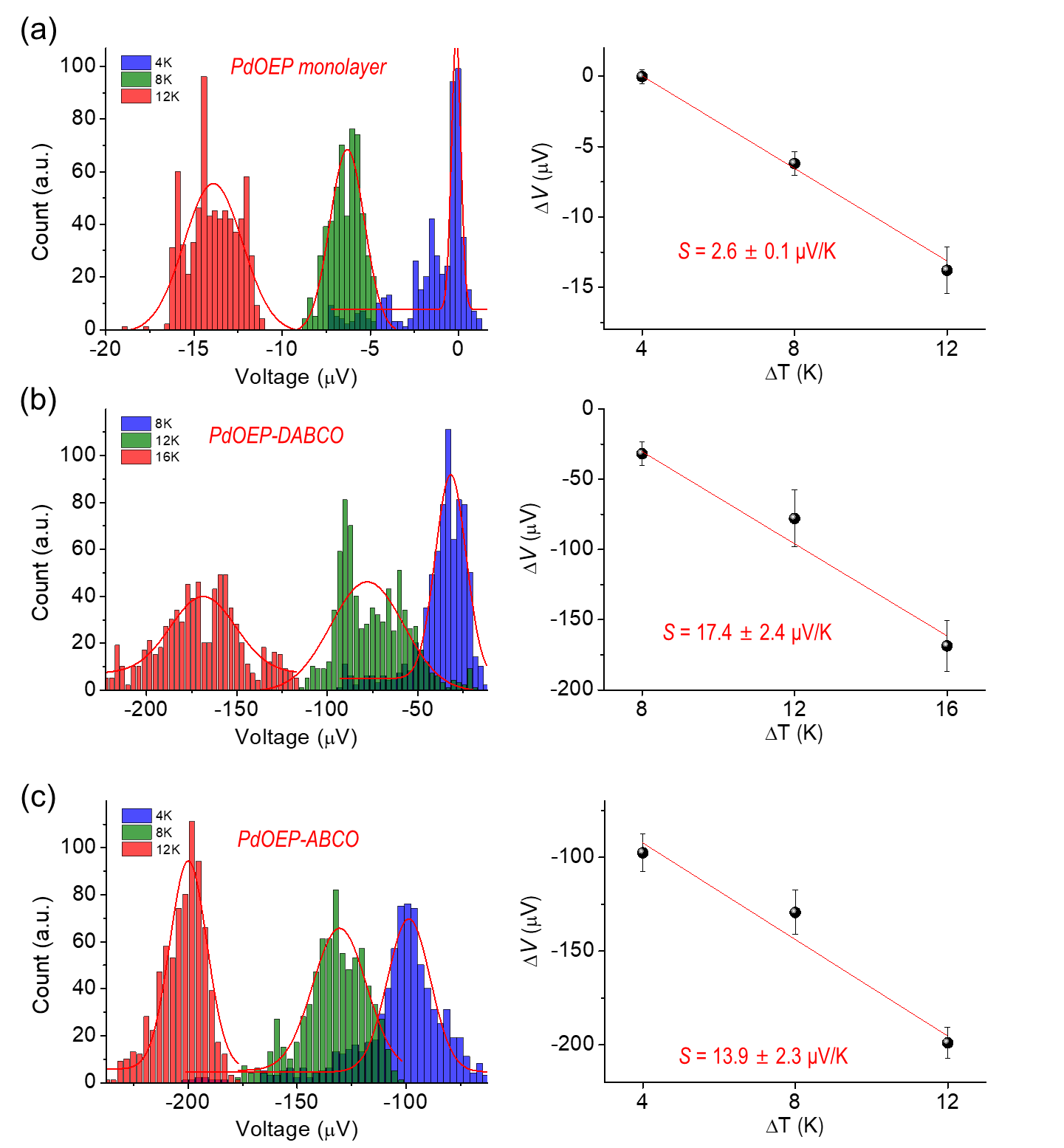
Figure S22.** Histograms of thermovoltage (Δ*V*, μV) and plots of Δ*V* as a function of Δ*T* for (a) PdOEP monolayer, (b) PdOEP-DABCO bilayer, and (c) PdOEP-ABCO bilayer junctions.

# References

[1] Q. Ferreira, A. M. Bragança, L. Alcácer, J. Morgado, *J. Phys. Chem. C* **2014**, *118*, 7229-7234.

[2] a) H.-W. Wang, C.-H. Chen, T.-S. Lim, S.-L. Huang, T.-Y. Luh, *Chem. Asian J.* **2011**, *6*, 524-533; b) L. Flamigni, A. M. Talarico, B. Ventura, R. Rein, N. Solladié, *Chem. - Eur. J.* **2006**, *12*, 701-712.

[3] a) J. Jang, J. W. Jo, T. Ohto, H. J. Yoon, *J. Am. Chem. Soc.* **2024**, *146*, 4922-4929; b) G. D. Kong, S. E. Byeon, J. Jang, J. W. Kim, H. J. Yoon, *J. Am. Chem. Soc.* **2022**, *144*, 7966-7971; c) P. He, A. H. Daaoub, S. Sangtarash, H. Sadeghi, H. J. Yoon, *Nano Lett.* **2024**, *24*, 1988-1995; d) J. Jang, P. He, H. J. Yoon, *Acc. Chem. Res.* **2023**, *56*, 1613-1622.

[4] J. M. Soler, E. Artacho, J. D. Gale, A. García, J. Junquera, P. Ordejón, D. Sánchez-Portal, *J. Condens. Matter Phys.* **2002**, *14*, 2745.

[5] a) J. Ferrer, C. J. Lambert, V. M. García-Suárez, D. Z. Manrique, D. Visontai, L. Oroszlány, R. Rodríguez-Ferradás, I. Grace, S. Bailey, K. Gillemot, *NJP* **2014**, *16*, 093029; b) H. Sadeghi, *Nanotechnology* **2018**, *29*, 373001.
